# Observation of temperature-gradient-induced magnetization

**DOI:** 10.1038/ncomms12265

**Published:** 2016-07-26

**Authors:** Dazhi Hou, Zhiyong Qiu, R. Iguchi, K. Sato, E. K. Vehstedt, K. Uchida, G. E. W. Bauer, E. Saitoh

**Affiliations:** 1 WPI Advanced Institute for Materials Research, Tohoku University, Sendai 980-8577, Japan; 2 Spin Quantum Rectification Project, ERATO, Japan Science and Technology Agency, Sendai 980-8577, Japan; 3 Institute for Materials Research, Tohoku University, Sendai 980-8577, Japan; 4 London Centre for Nanotechnology and Department of Electronic and Electrical Engineering, University College London, 17-19 Gordon Street, London WC1H 0AH, UK; 5 PRESTO, Japan Science and Technology Agency, Saitama 332-0012, Japan; 6 Kavli Institute of NanoScience, Delft University of Technology, Delft 2628 CJ, The Netherlands; 7 Advanced Science Research Center, Japan Atomic Energy Agency, Tokai 319-1195, Japan

## Abstract

Applying magnetic fields has been the method of choice to magnetize non-magnetic materials, but they are difficult to focus. The magneto-electric effect and voltage-induced magnetization generate magnetization by applied electric fields, but only in special compounds or heterostructures. Here we demonstrate that a simple metal such as gold can be magnetized by a temperature gradient or magnetic resonance when in contact with a magnetic insulator by observing an anomalous Hall-like effect, which directly proves the breakdown of time-reversal symmetry. Such Hall measurements give experimental access to the spectral spin Hall conductance of the host metal, which is closely related to other spin caloritronics phenomena such as the spin Nernst effect and serves as a reference for theoretical calculation.

## Abstract (web summary)

In general, heating increases disorder and leads to the loss of magnetism in condensed matter. Here, the authors demonstrate that a normal metal can be magnetized by applying a temperature gradient during non-uniform heating when attached to a magnetic insulator.

## Graphical abstract

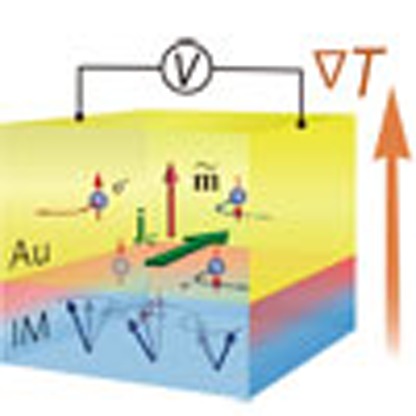

Generation and detection of non-equilibrium magnetization in non-magnetic conductors lie at the heart of spintronics research^1^,^2^,^3^. In previous studies, it was shown that spin injection can be achieved by various techniques^4^,^5^,^6^,^7^,^8^,^9^,^10^,^11^. However, the detection of the spin injection induced magnetization is more challenging because of its small magnitude. Especially, the non-equilibrium magnetization induced time-reversal symmetry breaking has been rigorously confirmed in very limited systems, which usually requires the observation of Faraday(Kerr)-type phenomena or Hall effect^7^,^12^,^13^,^14^. For some thermal spin injection methods such as the spin Seebeck effect^8^,^15^,^16^,^17^,^18^, the temperature-gradient-induced spin current can be detected by the inverse spin Hall effect (ISHE) while expected accompanying magnetization still lacks direct experimental evidence.

We start with considering a simple bilayer film comprising an insulator magnet such as Y_3_Fe_5_O_12_ (YIG) and a normal metal layer, such as Au (see Fig. 1a). Au is far from a ferromagnetic instability and the static proximity magnetization is negligibly small^8^,^19^. Perturbation, such as a temperature gradient, generates non-equilibrium magnetization dynamics in the magnet^4^,^20^,^21^. In such a system, non-equilibrium spin phenomena can be classified in terms of the following three regimes. First, when the spin diffusion length of the metal layer *l*_D_ is much less than its thickness *t*, *l*_D_<<*t*; the normal metal properties are affected only over a thin-skin layer next to the ferromagnet, that is, the trivial class. Second, when *l*_D_∼*t*, the magnetization dynamics excited in the magnet injects spin angular momentum deep into the metal layer. However, the decay of the spin accumulation is steep and its average is suppressed^22^. Most studies that detect spin Seebeck and spin-pumping effects by the ISHE are in this regime, in which a heat current and microwave-induced magnetization dynamics are treated as a perturbation, respectively.

Finally, in the regime, *l*_D_⩾*t*, the perturbation is able to magnetize the entire metal layer in a non-equilibrium manner as illustrated in Fig. 1a (ref. 23): non-equilibrium magnetization, which breaks time-reversal-symmetry. The created non-equilibrium magnetization 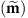 may induce a Hall electromotive force **E**_H_ when a probe electric current, **j**_c_, is applied as illustrated in Fig. 1b:


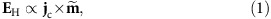


just as the equilibrium magnetization of ferromagnets induces the anomalous Hall effect (AHE)^24^,^25^,^26^,^27^. Observation of such a Hall effect has been considered difficult since non-equilibrium magnetization in metals is small and short-lived^13^.

In this article, we, nevertheless, report a non-equilibrium magnetization created by a temperature gradient observed with a lock-in Hall measurement at room temperature. In addition, we also show that the non-equilibrium magnetization generated by spin pumping can be measured by the same method. The Hall measurement method we developed enables the observation of such tiny magnetization, which highlights its high sensitivity and potential application in spintronics studies.

## Results

### Temperature-gradient-induced non-equilibrium magnetization

**Figure 2:**
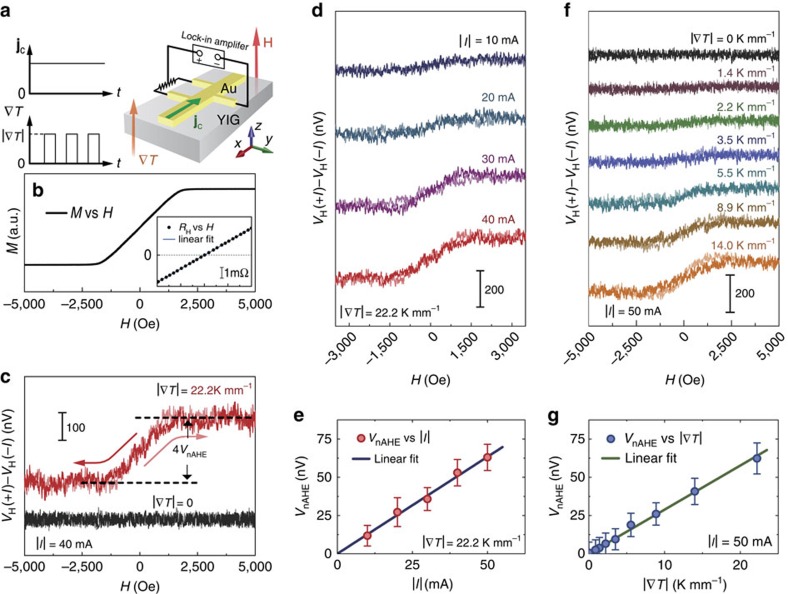
Temperature-gradient-induced magnetization. (**a**) An Illustration of the Hall measurement setup for the temperature-gradient-induced magnetization. A 20-nm-thick Au film on YIG is patterned into a Hall bar. A standard five-probe method was used for the Hall measurement. A magnetic field was applied perpendicular to the sample, which is of a 2 mm × 3 mm rectangle. A d.c. current, *I*, and an a.c. out-of-plane temperature gradient, ∇*T*, were applied simultaneously, while the Hall voltage was picked up with a lock-in amplifier. (**b**) Out-of-plane magnetization curve for the Au/YIG sample at 300 K. The inset shows a linear Hall response of the Au film at 300 K, indicating a negligible static magnetic proximity. (**c**) The Hall signal measured for |∇*T*|=22.2 K mm^−1^ (red curve) and zero (black curve) at |*I*|=40 mA. No magnetic-field response was observed in the latter case. Otherwise, the Hall signal shows an asymmetric magnetic-field dependence of the same saturation behaviour as the *M*-*H* curve in **b**. (**d**) Hall voltages measured for different current levels (|*I*|) at |∇*T*|=22.2 K mm^−1^. (**e**) The |*I*| dependence of the nAHE signal shown in **d**. The solid line is a linear fit. The error bar is estimated by the s.d. of the measured voltage above the saturation field. (**f**) The Hall voltage measured at various values of the temperature gradient. The nAHE signal vanishes when |∇*T*|=0 and increases with increasing |∇*T*|. (**g**) The |∇*T*| dependence of the nAHE signal shown in **f**. The solid line is a linear fit. The error bar is estimated in the same way as in **e**.

Figure 2a illustrates the experimental setup used in the present study. The first sample is a 20-nm-thick Au/5-μm-thick Y_3_Fe_5_O_12_ (YIG) bilayer film on a Gd_3_Ga_5_O_12_ substrate, where YIG is a ferrimagnetic insulator with the band gap of 2.6 eV. The YIG magnetization was aligned in the out-of-plane direction by an external magnetic field *H*>0.2 T (see *M*-*H* curve in Fig. 2b). The Hall effect in the Au film was measured using an in-plane current and an out-of-plane temperature-gradient ∇*T*, that is, a configuration that fulfills the conditions for the Hall effect anticipated above. On the other hand, a spin Seebeck voltage generated by the ISHE should vanish because of the symmetry. By simultaneously applying^28^ an a.c. temperature-gradient ∇*T* and d.c. in-plane current *I*, we selectively picked up the Hall voltage component *V*_H_ that is proportional to ∇*T* (see Methods for details), while ordinary Hall components are removed by the lock-in method. We eliminated heat-induced parasitic effects that do not depend on the current direction by recording the authentic Hall response *V*_H_(+*I*)−*V*_H_(−*I*).

First, in Fig. 2c, we show the Hall voltages *V*_H_(+*I*)−*V*_H_(−*I*) measured in the absence of a temperature gradient (∇*T*=0). In *V*_H_(+*I*)−*V*_H_(−*I*), no field dependent signal appears, consistent with the absence of a conventional AHE that would appear in a proximity magnetized Au. For the conventional spin Seebeck measurement please see Supplementary Figs 1 and 2, and Supplementary Note 1.

Figure 2c shows the Hall voltage *V*_H_(+*I*)−*V*_H_(−*I*) measured with applying a temperature-gradient, ∇*T*=22.2 K mm^−1^ and |*I*|=40 mA (red curves). Importantly, the measured Hall voltage exhibits AHE-like antisymmetric field dependence and the same saturation behaviour as the *M*-*H* curve in Fig. 2b, which manifests time-reversal symmetry breaking. The field dependence of the Hall voltage similar to the *M*-*H* curve suggests that 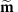 parallel to the YIG magnetization appears in the Au layer due to the temperature gradient.

Figure 2d shows a linear |*I*|-dependence of the Hall voltage at |∇*T*|=22.2 K mm^−1^, that vanishes at |*I*|→0 as shown in Fig. 2e, ruling out a contamination by the symmetry-forbidden ISHE detection of the spin Seebeck effect. Also, the observed field dependence of the Hall voltage can not be explained by the spin Hall magnetoresistance^29^, which has an even magnetic-field symmetry.

Figure 2f shows the temperature-gradient dependence of *V*_H_(+*I*)−*V*_H_(−*I*) at |*I*|=50 mA. The AHE-type signal is zero at |∇*T*|=0 as mentioned above and gradually emerges with increasing |∇*T*|. The data prove its non-equilibrium origin that distinguishes it from the AHE due to magnetic proximity^30^,^31^. Figure 2g demonstrates a linear relation between the Hall voltage and |∇*T*|; the temperature gradient over the Au/YIG bilayer breaks time-reversal symmetry in the Au film: an essential feature of a spin polarization. In other words, the observed Hall voltage is evidence of a temperature-gradient-induced magnetization in Au. We call this phenomenon a non-equilibrium AHE (nAHE).

### Spin-pumping-induced non-equilibrium magnetization

**Figure 3:**
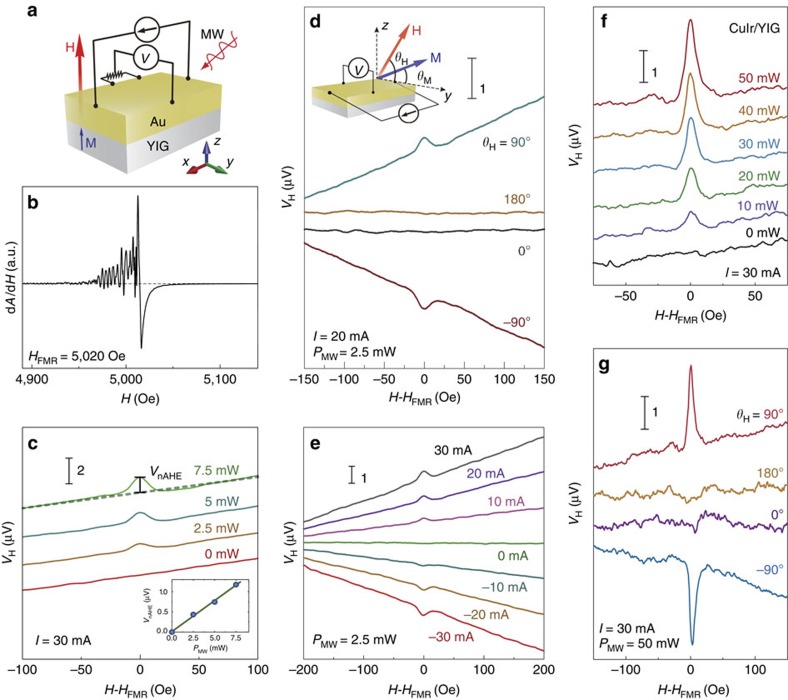
Spin-pumping-induced magnetization. (**a**) An illustration of the Hall measurement setup for spin-pumping induced magnetization. The sample is a 14-nm-thick Au/YIG bilayer film without patterning. A continuous microwave and a current were applied to the sample while a d.c. Hall voltage was picked up. The sample was placed at the centre of a TE_011_ microwave (MW) cavity with the resonance frequency 9.45 GHz. The microwave magnetic field is along the *x* axis. (**b**) Field (*H*) dependence of the FMR signal (d*A*/d*H*) under 1 mW microwave excitation, *A* being the microwave absorption. The ferromagnetic resonance field is *H*_FMR_=5,020 Oe. (**c**) Field dependence of the Hall voltage at different values of the microwave power. The sensing current is fixed at 30 mA. The inset is the microwave power *P*_MW_ dependence of the generated Hall voltage in the Au *V*_nAHE_. The solid line is a linear fit. (**d**) The Hall voltage measured with in-plane (*θ*_H_=0°, 180°) and perpendicular (*θ*_H_=90°, −90°) magnetic fields at 2.5 mW microwave excitation and *I*=30 mA. The inset shows the definition of the magnetic-field angle *θ*_H_ and the magnetization angle *θ*_M_. (**e**) Current magnitude dependence of the Hall voltage measured at *P*_MW_=2.5 mW. (**f**) Hall voltage measured in a 24-nm-Cu_95_Ir_5_/YIG film at different values of microwave power with the same setup as in **a**. (**g**) The field angle dependence of the Hall signal in a Cu_95_Ir_5_/YIG film.

To double check this scenario, for a similar film, we also measured nAHE induced by deterministic spin pumping, in which the magnetization dynamics is resonantly excited by microwaves as illustrated in Fig. 3a, rather than a temperature gradient. We observed nAHE as well as an ordinary Hall effect as a d.c. voltage that appears when simultaneously applying continuous microwaves and a d.c. current.

Figure 3b shows a microwave absorption spectrum near the ferromagnetic resonance (FMR) with out-of-plane magnetic fields. In Fig. 3c, the Hall voltage is measured around the FMR field *H*_FMR_ at different values of microwave power for the same applied current. At zero microwave power (*P*_MW_=0), we only observe the ordinary Hall voltage that increases linearly with field.. When a microwave is applied, on the other hand, a conspicuous signal proportional to *P*_MW_ emerges at *H*_FMR_ on top of the normal Hall effect. The peak voltage as a function of the out-of-plane magnetic-field angle *θ*_H_ is shown in Fig. 3d; its sign changes by reversing the field direction and it vanishes for in-plane magnetic fields (*θ*_H_=0 and 180°), consistent with the nAHE scenario.

The voltage peak is not caused by the ISHE induced by the spin pumping. By switching off the d.c. current *I*, the peak voltage disappears as shown by the green curve in Fig. 3e; indeed the ISHE should not appear because of the symmetry of the perpendicular magnetized configuration. The result indicates that the simultaneous application of the microwave and the current is necessary for the present Hall voltage to appear. Figure 3e shows the Hall voltage for various values of the current under the microwave irradiation. Both the normal Hall and the peak components are found to be proportional to the current amplitude and change their signs with reversing the current polarity. Thus, *I*-independent mechanisms such as ISHEs^4^ and magnetoelectric rectification^32^,^33^ are ruled out. The peak voltage is thus attributed to the nAHE, reinforcing its concept of nAHE.

We also observed nAHE signals in another paramagnetic metal: a Cu_95_Ir_5_ film on YIG, which shows a similar microwave-power and field-angle dependence, as shown in Fig. 3f,g, respectively. The nAHE appears to be universal, and it should be worthwhile to explore it in other material systems. The measurement of this effect is free of ferromagnetic electrode and does not require nanofabrication, of which the device structure is much simpler compared with previous techniques for thermal induced spin accumulation observation^34^,^35^,^36^,^37^,^38^.

## Discussion

We formulate the nAHE in terms of the spectral spin Hall effect (SHE). The non-equilibrium magnetization in a normal metal is proportional to the spin accumulation: *μ*_s_=*μ*^↑^−*μ*^↓^, while the total charge density and Fermi energy remain to leading order unmodified. 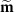 is parallel to the minority spin direction in the paramagnet. When a longitudinal electric field (**E**) is applied, Hall currents are induced via the spin–orbit interaction^39^,^40^: 
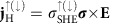
, where 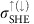 is the spin Hall conductivity for up (down) spin electrons and ***σ*** is the electron spin polarization vector. For small *μ*_s_, 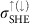 can be expanded as 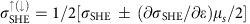, in which *σ*_SHE_ is the ground-state spin Hall conductivity and the derivative is taken at the Fermi energy. The total Hall current reads:


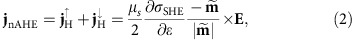


According to equation (2), the nAHE current in the linear response of *μ*_s_ is proportional to the energy derivative of the spin Hall conductivity (*∂σ*_SHE_/*∂ɛ*), while the spin Hall and anomalous Hall effects scale with *σ*_SHE_ and *σ*_AHE_, respectively^41^,^42^,^43^.

*∂σ*_SHE_/*∂ɛ* can be decomposed as


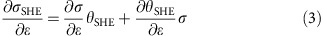


where the spin Hall angle *θ*_SHE_=*σ*_SHE_/*σ* and *σ* is the longitudinal conductivity of a normal metal film. The second-term proportional to *∂θ*_SHE_/*∂ɛ* in equation (3) represents the Hall current generated by the difference between the spin Hall angles of the up- and down-spin electrons. The first term is estimated to account for only one per cent of the observed nAHE voltage (see Supplementary Fig. 3, and Supplementary Notes 2 and 3), which suggests that the *∂θ*_SHE_/*∂ɛ* term dominates the signal. A significant energy dependence of *θ*_SHE_ has recently been predicted and observed in some metals^44^,^45^,^46^,^47^,^48^.

In summary, we show that Au is magnetized by applying a temperature gradient or a microwave on YIG/Au bilayers. Our work also highlights the electron transport property modified by spin injection and thus offers a versatile approach for the generation and detection of non-equilibrium magnetizations in normal metals.

## Methods

### Sample fabrication

The single-crystaline yttrium iron garnet (YIG) films used in the present work were prepared by a liquid phase epitaxy on a gallium gadolinium garnet substrate. All samples were cut from a single 4.5-μm-thick liquid phase epitaxy YIG film. To achieve good-YIG/metal interfaces, the YIG films underwent an acid pickling process before being transferred into high vacuum where they were annealed *in situ* for 3 h at 500 °C. Then, the normal metal layers were sputtered at room temperature. The thickness of Au and Cu_95_Ir_5_ films was calibrated by X-ray reflectometry using control samples. The crystallographic properties of the YIG/Cu_95_Ir_5_ device were characterized by transmission electron microscopy and X-ray diffractometry, to confirm that the YIG films were high quality single crystals with a lattice constant of 12.376 Å.

### Measurement setup

In the temperature-gradient-induced magnetization measurements, we used a microwave-induced heating method in off-resonance conditions. The Au/YIG sample was placed on a coplanar waveguide, and an a.c. temperature gradient was induced by applying a pulsed 20 GHz microwave with the modulation frequency 31.28 Hz. The applied magnetic-field well resided in the off-resonance range of the YIG layer. Therefore, the microwave causes no FMR, but induces heating of the Au layer via electromagnetic induction. The a.c. temperature gradient near the Au/YIG interface was confirmed by an a.c. spin Seebeck measurement and calibrated by the spin Seebeck voltages measured with a d.c. temperature gradient. Under an a.c. temperature gradient, an applied d.c. current generates a d.c. ordinary Hall signal and an a.c. nAHE signal, in which only the a.c. nAHE signal can be captured by a lock-in amplifier. For the Hall measurement, a standard five-probe method was used, in which a potentiometer is adopted to compensate the longitudinal voltage drop mixed in the Hall signal, and the out-of-plane alignment of magnetic field is confirmed in the absence of the spin Seebeck effect at saturated magnetization.

### Data availability

All relevant data are available on request, which should be addressed to D.H.

## Additional information

**How to cite this article:** Hou, D. *et al*. Observation of temperature-gradient-induced magnetization. *Nat. Commun.* 7:12265 doi: 10.1038/ncomms12265 (2016).

## Supplementary Material

Supplementary Information Supplementary Figures 1-3, Supplementary Notes 1-3 and Supplementary References

Peer Review File

## Figures and Tables

**Figure 1:**
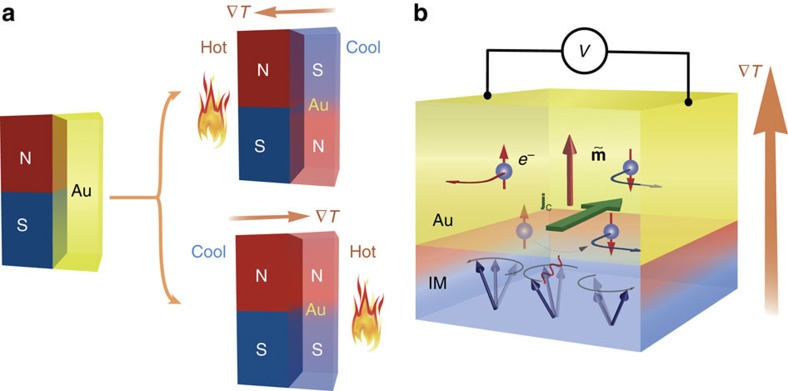
Concept of the non-equilibrium magnetization. (**a**) In a normal metal/insulator magnet film, a non-equilibrium magnetization 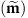 can be generated in the normal metal (for example, Au) via a dynamic spin exchange by external perturbations such as a temperature-gradient (∇*T*). The orientation of 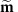 is related to the direction of the applied ∇*T*. (**b**) In the presence of a non-equilibrium magnetization, spin-up and spin-down electrons are unequally populated. When a charge current (**j**_c_) is applied normal to 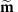, an anomalous Hall-type voltage is expected.
