# Supplementary material for: Observation of temperature-gradient-induced magnetization
Source: Nat Commun. 2016 Jul 26;7:12265. doi: 10.1038/ncomms12265 (PMC4963471; doi:10.1038/ncomms12265)
Supplement: Supplementary Information — Supplementary Figures 1-3, Supplementary Notes 1-3 and Supplementary References [file ncomms12265-s1.pdf]

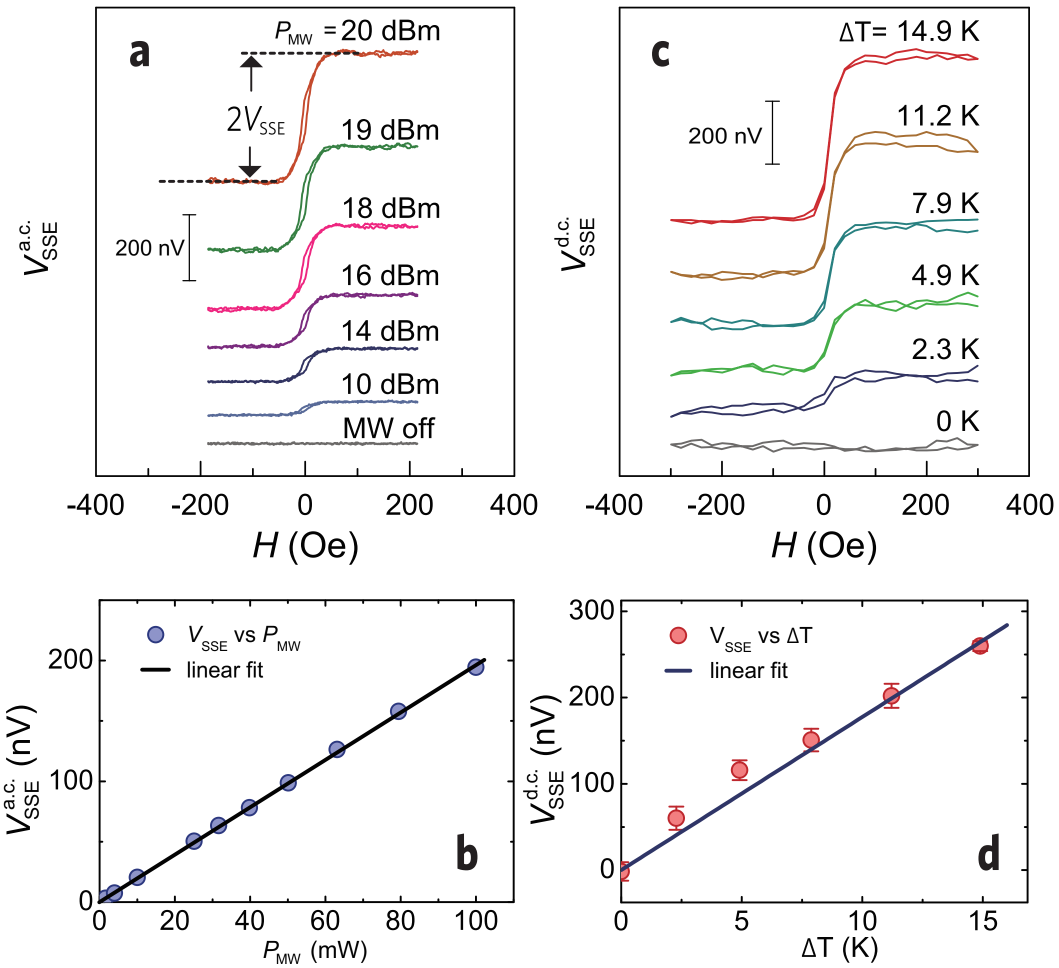

Supplementary Figure 1: **The longitudinal spin Seebeck effect measured by a.c. and d.c. temperature gradients.** Error bars are estimated by the standard deviation.

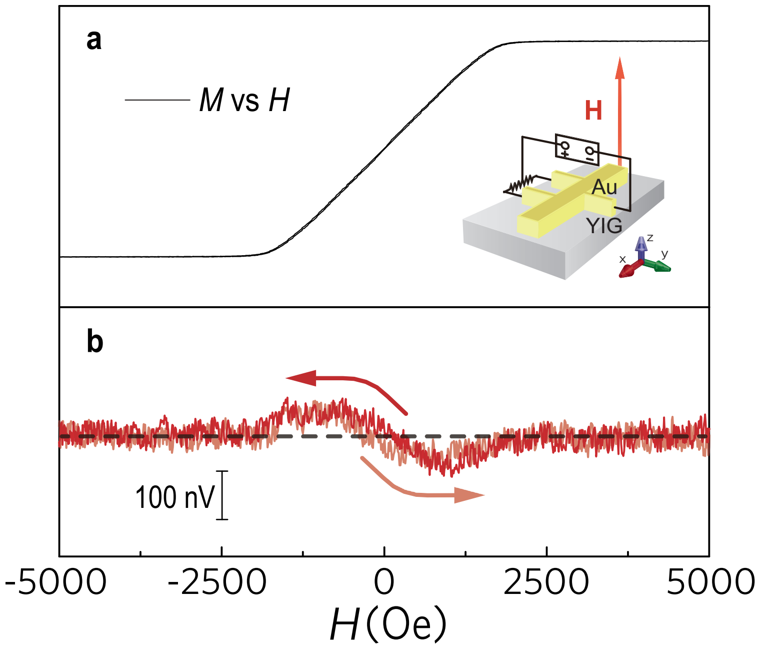

Supplementary Figure 2: **Out-of-plane field M-H curve and the corresponding spin Seebeck signal.** The spin Seebeck signal shows some finite value before saturation of magnetization because of in-plane magnetization component, and vanishes after saturation, which obeys the symmetry of spin Seebeck effect and excludes the possible spin Seebeck contamination in our Hall measurement.

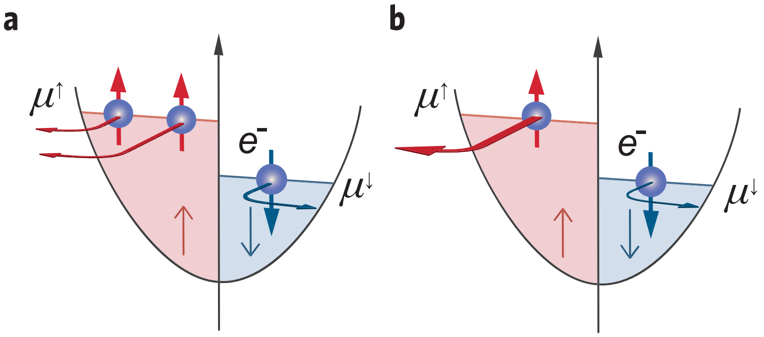

Supplementary Figure 3: **Two different scenarios of the Hall current generation.** **a**, A Hall current is generated due to the number difference between up- and down-spin electrons with a same spin Hall angle. This scenario corresponds to the first term in Supplementary Equation (8). **b**, A Hall current is caused by the spin Hall angle difference between up- and down-spin electrons. This new scenario corresponds to the second term in Supplementary Equation (8).

Supplementary Note 1: calibration of the microwave-induced temperature gradient at Au/YIG interface

The a.c longitudinal spin Seebeck effect induced by microwave heating was measured with an in-plane magnetic field. The data is shown in Supplementary Figure 1a. The applied microwave was modulated in a pulsed mode[1] . The spin Seebeck voltage shows a linear dependence on the applied microwave power P_{MW}, as shown in Supplementary Figure 1b, indicating P_{MW} is proportional to the temperature gradient at the Au/YIG interface. To calibrate the microwave-heating induced temperature gradient, we measured the longitudinal spin Seebeck effect in the same sample with a d.c. temperature gradient which is generated with two heat baths between which temperature difference ΔT is applied. The d.c. spin Seebeck signal is shown in Supplementary Figure 1c and the ΔT dependence is shown in Supplementary Figure 1d. With the GGG substrate thickness of 0.5 mm, which is much larger than that of the YIG (5 μm) and Au (20 nm) layer, we calculate d.c. temperature gradient. Then by comparing the spin Seebeck signal magnitudes in the a.c. and d.c. measurements, we can obtain the ratio between the a.c. temperature gradient at Au/YIG interface and the applied microwave power: $\nabla T_{a.c.}/P_{\mathrm{MW}} = 0.222$ (K/mm)/mW. The spin Seebeck effect is also measured at the out-of-plane magnetic field setup as in Supplementary Figure 2, which clear shows that the spin Seebeck signal is well suppressed below the error bar of our measurement.

Supplementary Note 2: spin accumulation in the paramagnetic layer

In this section, we model the d.c. spin accumulation in the paramagnetic metal layer due to the spin pumping from the YIG layer. The z axis is normal to the film and the metal/YIG interface is at $z = 0$. The d.c. spin accumulation $\mu_{\mathrm{s}}(z) \equiv \mu^{\uparrow}(z) - \mu^{\downarrow}(z)$ is governed by the

spin diffusion equation,[2]

$$\frac{\partial^2}{\partial z^2}\mu_\mathrm{s}(z) = \frac{1}{\lambda_\mathrm{s}^2}\mu_\mathrm{s}(z) \tag{1}$$

where λ_s denotes the spin-flip diffusion length. The diffusion spin current density is $j_\mathrm{s}(z) = -\frac{\hbar}{4e^2}\sigma\frac{\partial}{\partial z}\mu_\mathrm{s}$, where σ denotes the charge conductivity. The boundary conditions are $j_\mathrm{s}(0) = j_\mathrm{s}^\mathrm{pump} - j_\mathrm{s}^\mathrm{back}$ at the interface ($z = 0$) and $j_\mathrm{s}(d) = 0$ at the surface of the metal layer ($z = d$), where $j_\mathrm{s}^\mathrm{pump}$ and $j_\mathrm{s}^\mathrm{back}$ denote pumped and back-flow spin current densities, respectively.[3] At the interface[3]

$$j_\mathrm{s}^\mathrm{pump} = \frac{\hbar}{4\pi M_\mathrm{s}^2} g_r^{\uparrow\downarrow} \left|\left\langle \mathbf{M} \times \dot{\mathbf{M}} \right\rangle_t\right|, \tag{2}$$

where $\mathbf{M}$ denotes the magnetization in the YIG layer, M_s the saturation magnetization, $g_r^{\uparrow\downarrow}$ the real part of the spin mixing conductance per unit area, and $\langle\rangle_t$ the time average. In Au and $\mathrm{Cu_{95}Ir_5}$, spin-flip scattering is sufficiently efficient that d.c. component of the back-flow spin current density reduces to[3]

$$j_\mathrm{s}^\mathrm{back} = \frac{1}{4\pi} g_r^{\uparrow\downarrow} \mu_\mathrm{s}(0). \tag{3}$$

Using the above boundary conditions for Supplementary Equation (1), we obtain an averaged spin accumulation

$$\bar{\mu_\mathrm{s}} \equiv \frac{1}{d}\int_0^d \mu_\mathrm{s}(z)\,dz = \frac{\lambda_\mathrm{s}^2}{dM_\mathrm{s}^2} \frac{e^2\hbar g_r^{\uparrow\downarrow}\left|\left\langle \mathbf{M} \times \dot{\mathbf{M}} \right\rangle_t\right|}{\pi\hbar\sigma + e^2\lambda_\mathrm{s} g_r^{\uparrow\downarrow}\coth(d/\lambda_\mathrm{s})}. \tag{4}$$

The spin accumulation in Au and $\mathrm{Cu_{95}Ir_5}$ induced by spin pumping can be estimated by using Supplementary Equation (4) with the relevant parameters: damping constant of YIG film α_YIG=1× 10^{-4}, $4\pi M_\mathrm{s}$= 1617 Oe, Au film conductivity σ_Au= 1.5 $\times 10^7$ $\Omega^{-1}\mathrm{m}^{-1}$, spin diffusion length λ_Au= 60 nm, Au film thickness d_Au= 14 nm, Au/YIG interface spin mixing conductance $g_\mathrm{YIG/Au}^{\uparrow\downarrow}$= 5 $\times 10^{18}/\mathrm{m}^2$ and microwave field h_ac= 0.3 Oe at 7.5 mW,[4–6] we obtain the spin accumulation in Au: $\bar{\mu_\mathrm{s}}|_{7.5\mathrm{mW}}^{\mathrm{Au}}$= 3.6 μeV. With $g_\mathrm{YIG/CuIr}^{\uparrow\downarrow}$= 8.4 $\times 10^{18}/\mathrm{m}^2$, $\lambda_{\mathrm{Cu_{95}Ir_5}}$= 24 nm determined by a $\mathrm{Cu_{95}Ir_5}$ thickness dependence study and $\sigma_{\mathrm{Cu_{95}Ir_5}}$= 3.8$\times 10^6$ $\Omega^{-1}\mathrm{m}^{-1}$,

we obtain the spin accumulation in 24 nm $Cu_{95}Ir_5$ film $\bar{\mu_s}|^{Cu_{95}Ir_5}_{50mW} = 19\ \mu$eV.

Supplementary Note 3: the non-equilibrium anomalous Hall resistance

According to Supplementary Equation (2) in the main text, the nAHE current generated in the metal layer is:

$$I_{\mathrm{nAHE}} = \int_0^d dI_{\mathrm{nAHE}}(z) = \frac{ld}{2}\frac{\partial\sigma_{\mathrm{SHE}}}{\partial\varepsilon}\bar{\mu_{\mathrm{s}}}\frac{-\widetilde{\mathbf{m}}}{|\widetilde{\mathbf{m}}|}\times\mathbf{E}\cdot\hat{\mathbf{y}} \tag{5}$$

in which l is the length of the sample in the current direction and $\bar{\mu_s}$ the spin accumulation Supplementary Equation (4). The Hall voltage in an open circuit compensates the Hall current in Supplementary Equation (5):

$$V_{\mathrm{nAHE}} = -\frac{wI_{\mathrm{nAHE}}}{ld(\sigma^{\uparrow}+\sigma^{\downarrow})} \tag{6}$$

where w is the width of the sample in the transverse direction and $\sigma^{\uparrow(\downarrow)}$ denotes the electric conductivity in the spin-up (spin-down) subband. To the first order of μ_s, $\sigma^{\uparrow(\downarrow)} = \sigma/2 \pm (\mu_s/4)\partial\sigma/\partial\varepsilon$, and $\sigma^{\uparrow}+\sigma^{\downarrow} = \sigma$. From Supplementary Equation (6) we obtain the nAHE resistance:

$$R_{\mathrm{nAHE}} = \frac{V_{\mathrm{nAHE}}}{I} = -\frac{1}{2\sigma^2 d}\frac{\partial\sigma_{\mathrm{SHE}}}{\partial\varepsilon}\bar{\mu_{\mathrm{s}}}\sin\theta_{\mathrm{M}} \tag{7}$$

in which I denotes the longitudinal current. According to the nAHE in the spin pumping experiments, $R^{\mathrm{Au}}_{\mathrm{nAHE}}|_{7.5mW} = 39\ \mu\Omega$, $R^{Cu_{95}Ir_5}_{\mathrm{nAHE}}|_{50mW} = 81\ \mu\Omega$, and the spin accumulation value calculated in last section, we obtain $\partial\sigma^{\mathrm{Au}}_{\mathrm{SHE}}/\partial\varepsilon = -68300\ \Omega^{-1}\mathrm{m}^{-1}$/meV and

$\partial\sigma^{Cu_{95}Ir_5}_{\mathrm{SHE}}/\partial\varepsilon = -2950\ \Omega^{-1}\mathrm{m}^{-1}$/meV.

$\partial\sigma_{\mathrm{SHE}}/\partial\varepsilon$ can be decomposed into two terms:

$$\frac{\partial\sigma_{\mathrm{SHE}}}{\partial\varepsilon} = \frac{\partial\sigma}{\partial\varepsilon}\theta_{\mathrm{SHE}} + \frac{\partial\theta_{\mathrm{SHE}}}{\partial\varepsilon}\sigma \tag{8}$$

where we define the spin Hall angle as $\theta_{\mathrm{SHE}} = \sigma_{\mathrm{SHE}}/\sigma$ and σ is the conductivity of the $Cu_{95}Ir_5$ film. The first term in Supplementary Equation (8) corresponds to the standard picture of the anomalous Hall effects in ferromagnets, which attribute the Hall current to the number difference between majority and minority electrons, as illustrates in Supplementary Figure 3a. Since $\partial\sigma/\partial\varepsilon = S/eL_0T$ (S is the Seebeck coefficient, L_0 the Lorenz number),[7] we estimate $\theta_{\mathrm{SHE}}^{\mathrm{Cu_{95}Ir_5}}\partial\sigma/\partial\varepsilon = -35\ \Omega^{-1}\mathrm{m}^{-1}/\mathrm{meV}$ with $S_{\mathrm{Cu_{95}Ir_5}} = 3.5\ \mu\mathrm{V/K}$ as measured in the $Cu_{95}Ir_5$ film and $\theta_{\mathrm{SHE}}^{\mathrm{Cu_{95}Ir_5}} = -0.02$,[8] and $\theta_{\mathrm{SHE}}^{\mathrm{Au}}\partial\sigma/\partial\varepsilon = -306\ \Omega^{-1}\mathrm{m}^{-1}/\mathrm{meV}$ with $S_{\mathrm{Au}} = 1.5\ \mu\mathrm{V/K}$ and $\theta_{\mathrm{SHE}}^{\mathrm{Au}} = -0.1$.[9] The first term in Supplementary Equation (8) is too small to account for the observed nAHE signal. It indicates that the second term in Supplementary Equation (8), which corresponds to another scenario of the Hall current generation as illustrated in Supplementary Figure 3b, dominates the nAHE signal. We can obtain the energy derivative of the spin Hall angle: $\partial\theta_{\mathrm{SHE}}^{\mathrm{Au}}/\partial\varepsilon$= -4.53/eV and $\partial\theta_{\mathrm{SHE}}^{\mathrm{Cu_{95}Ir_5}}/\partial\varepsilon$ = -0.77/eV. $\partial\theta_{\mathrm{SHE}}^{\mathrm{Cu_{95}Ir_5}}/\partial\varepsilon$ can be semi-quantitatively understood with a recent calculation on resonant skew scattering in $Cu_{95}Ir_5$,[10] while more theoretical efforts are needed to understand the value of $\partial\theta_{\mathrm{SHE}}^{\mathrm{Au}}/\partial\varepsilon$. It should be noted that the spin Hall effect dispersion in W, Pt, and Ir have been studied by spin Hall effect tunneling spectroscopy,[11] which indicates significant energy dependence of the spin Hall angle may exist in many materials.

Supplementary References

(1) Agrawal, M. et al. Microwave-induced spin currents in ferromagnetic-insulator—normal-metal bilayer system. Applied Physics Letters **105**, 092404 (2014).

(2) Johnson, M. & Silsbee, R. H. Spin-injection experiment. Phys. Rev. B **37**, 5326–5335 (1988).

(3) Tserkovnyak, Y., Brataas, A. & Bauer, G. E. W. Enhanced Gilbert damping in thin ferromagnetic films. Phys. Rev. Lett. **88**, 117601 (2002).

(4) Takahashi, R. et al. Electrical determination of spin mixing conductance at metal/insulator interface using inverse spin hall effect. Journal of Applied Physics **111** (2012).

(5) Burrowes, C. B. Enhanced spin pumping at yttrium iron garnet/Au interfaces. Appl. Phys. Lett. **100**, 92403 (2012).

(6) Ando, K. et al. Inverse spin-Hall effect induced by spin pumping in metallic system. Journal of Applied Physics **109**, 103913 (2011).

(7) Ashcroft, N. & Mermin, N. Solid State Physics (Cengage Learning, 2011).

(8) Niimi, Y. et al. Extrinsic spin hall effect induced by iridium impurities in copper. Phys. Rev. Lett. **106**, 126601 (2011).

(9) Seki, T. Giant spin Hall effect in perpendicularly spin-polarized FePt/Au devices. Nature Mater. **7**, 125–129 (2008).

(10) Xu, Z., Gu, B., Mori, M., Ziman, T. & Maekawa, S. Sign change of the spin hall effect due to electron correlation in nonmagnetic cuir alloys. Phys. Rev. Lett. **114**, 017202 (2015).

(11) Liu, L., Chen, C.-T. & Sun, J. Z. Spin Hall effect tunnelling spectroscopy. Nature Physics **10**, 561–566 (2014).
