# Supplementary material for: Observation of temperature-gradient-induced magnetization
Source: Nat Commun. 2016 Jul 26;7:12265. doi: 10.1038/ncomms12265 (PMC4963471; doi:10.1038/ncomms12265)
Supplement: Peer Review File [file ncomms12265-s2.pdf]

Editorial Note: this manuscript has been previously reviewed at another journal that is not operating a transparent peer review scheme. This document only contains reviewer comments and rebuttal letters for versions considered at Nature Communications.
